# British Medicinal Plants

**Published:** 1892-06

**Authors:** Alfred Heath

**Affiliations:** London, England


### BRITISH MEDICINAL PLANTS.

Alfred Heath, M. D., F. L. S., London, England.

### Order 37.—Umbelliferæ (Continued).

*Conium Maculatum* (Hemlock).—The common hemlock is a very poisonous plant; but it is also a marvelous medicine when properly used. Baron Stoerck is said to have been the first that brought it into repute, on account of its extraordinary efficacy in curing scirrhus and other forms of cancers, as well as other diseases supposed to be incurable. It has been used with success in allaying the irritation and improving the appearance of ill-conditioned sores. It has been successfully used in chronic rheumatism, glandular swellings, and various periodical pains. It has been found of *singular* use in whooping-cough; applied externally it allays the pains of open and ulcerated cancers. It is said to be excellent applied externally against *hard swellings*, gout, rheumatism, and stiff joints. There is a good proving of *Conium.* It *produces* on the healthy violent stitches in the chest and breasts, as if a knife were plunged in, throbbing, inflammation, tenderness, and hardness of the mammary glands. It has repeatedly removed lumps and tumors from the breast and other parts, caused by blows. It has a very powerful action on the glands. It produces heaviness and weariness in all the limbs; bruised, paralyzed feelings in the limbs; numbness; pains in all the joints, as if beaten; bone pains; loss of power in lower limbs; great weakness.

It produces dry, tickling, spasmodic cough; violent attacks of cough, with great oppression, almost amounting to suffocation; cough causing whooping, worse in the night. The hemlock is one of the most valuable remedies in the treatment of both simple and malignant swellings, and the homœopathic proving of the plant shows that it has a close affinity with these conditions, as it produces very similar effects. Some years ago a gentleman brought his son to me with a large tumor on his knee caused by a cricket ball. The swelling had been treated for a considerable time by an allopath. Iodine was painted on, and other things done without the least effect. I was led to give Conium$^{30}$, and in a few weeks the tumor entirely disappeared. Shortly after, the gentleman met the Doctor and told him the lump had gone. "Oh! I am so delighted to hear it," said he. "Yes, I took him to a homœopath. Homœopathy cured it!" "Nonsense, my dear sir, it was simply that Nature reasserted itself." "Well," said the father, "if Homœopathy helps Nature to reassert itself, in future I am going to be a homœopath." And he has been ever since.

### Order 38.—Hederaceæ.

*Hedera Helix* (the common Ivy).—The ivy has been used against the atrophy of children. The leaves are often applied to running sores, and to cleanse and heal old ulcers, and they are said to destroy vermin in children's heads. The berries are purging and will also produce vomiting. They are said to be excellent against rheumatism and pains of all kinds.

Ivy is believed to be good in spleen troubles. It was used with some success in the plague of London and was believed to be alexipharmic; a decoction of the juice dropped into the ear is said to relieve toothache of the *other* side. It is from the stalk of this tree that the resinous juice called *Gummi-hederæ* exudes, which possesses corroborant, astringent, and anti-spasmodic virtues. I was once told by an allopathic doctor that he used a decoction of the berries to remove the effect of intoxicating liquors. I am sorry to say there is no proving of this drug, as I feel sure that it is a valuable one. It has been many times used experimentally of late years by some homœopaths. If any one will prove it I will send them some tincture or gum.

